# Freisetzung von Aluminium aus Glitzerpartikeln bei herausnehmbaren kieferorthopädischen Apparaturen

**DOI:** 10.1007/s00103-021-03361-6

**Published:** 2021-06-11

**Authors:** Lena Wepner, Harald Andreas Färber, Anna Weber, Andreas Jaensch, Ludger Keilig, Florian Andreas Heuser, Christoph Peter Bourauel

**Affiliations:** 1 Oralmedizinische Technologie, Zentrum für Zahn‑, Mund- und Kieferheilkunde, Medizinische Fakultät, Universitätsklinikum Bonn (AöR), Universität Bonn, Bonn, Deutschland; 2 Institut für Hygiene und öffentliche Gesundheit, Medizinische Fakultät, Universitätsklinikum Bonn (AöR), Universität Bonn, Venusberg-Campus 1 (Gebäude 63), 53127 Bonn, Deutschland; 3 Poliklinik für Zahnärztliche Prothetik, Propädeutik und Werkstoffwissenschaften, Zentrum für Zahn-, Mund- und Kieferheilkunde, Medizinische Fakultät, Universitätsklinikum Bonn (AöR), Universität Bonn, Bonn, Deutschland

**Keywords:** Lose Zahnspangen, Korrosion, Speichel, Migration, Kieferorthopädie, Loose braces, Corrosion, Saliva, Migration, Orofacial orthopaedics

## Abstract

**Hintergrund und Ziel:**

Um bei kieferorthopädischen Behandlungen die Therapietreue von Kindern zu unterstützen, werden bei herausnehmbaren Apparaturen häufig Glitzerpartikel in den Kunststoff eingebettet, die Aluminium (Al) enthalten. Bei einer Tragedauer von bis zu 16 h täglich über 2–3 Jahre kann angenommen werden, dass über die Zeit Al-Ionen in den Speichel diffundieren. Ziel der Studie war es, die Freisetzung von Al-Ionen aus dem Kunststoff unter Verwendung verschiedener kieferorthopädischer Drähte zu untersuchen.

**Materialien und Methode:**

Es wurden Prüfkörper (Oberfläche 5,65 cm^2^) aus kieferorthopädischem Kunststoff und verschiedenen Drähten angefertigt; die Hälfte enthielt Glitzerpartikel aus Aluminium. Die Prüfkörper wurden 7 Tage lang in Petrischalen mit 50 ml Korrosionsmedium (pH 2,3) gem. DIN EN ISO 10271 bei 37 °C eingelegt. Zur Quantifizierung der spezifischen Ionen in der Korrosionslösung wurde die induktiv gekoppelte Plasmamassenspektrometrie (inductively coupled plasma - mass spectrometry, ICP-MS) verwendet.

**Ergebnisse:**

Die statistische Analyse zeigte einen signifikanten Unterschied in der Konzentration der Al-Ionen zwischen Proben mit und ohne Glitzerpartikel. Die Konzentrationen aus Proben mit Glitzer erreichten bis zu 14.474 μg/l Al-Ionen, Proben ohne Glitzer enthielten im Durchschnitt 1260 μg/l. Ein geringer Anteil der Al-Ionen kann aus den Legierungen der Drähte stammen.

**Schlussfolgerungen:**

Es sollte untersucht werden, ob die Aluminiumkonzentration zu Gesundheitsrisiken für den Menschen führen kann. Angesichts der Befunde sollten Kieferorthopäden keine glitzerhaltigen Apparaturen anbieten, um die Aluminiumaufnahme mit dem Speichel zu minimieren. Es muss geklärt werden, ob die in der Mundhöhle vorgefundenen Bedingungen zu gleichen Ergebnissen führen wie unter den oben genannten. Gesetzliche Regelungen sollten entwickelt werden, um die Freisetzung von Aluminium aus kieferorthopädischen Produkten zu begrenzen.

## Abstract (en)

**Background and aim:**

In order to support children’s compliance with orthodontic treatment, glitter particles containing aluminium (Al) are often embedded in the acrylic of removable appliances. When worn for up to 16 h daily for 2–3 years, it can be assumed that Al ions diffuse into saliva over time. The aim of this study was to investigate the release of Al ions from the acrylic using different orthodontic wires.

**Materials and method:**

Test specimens (surface area 5.65 cm^2^) were prepared from orthodontic resin and various wires; half contained aluminium glitter particles. The test specimens were placed in Petri dishes containing 50 ml of corrosion medium (pH 2.3) according to DIN EN ISO 10271 at 37 °C for 7 days. Inductively coupled plasma mass spectrometry (ICP-MS) was used to quantify the specific ions in the corrosion solution.

**Results:**

Statistical analysis showed a significant difference in the concentration of Al ions between samples with and without glitter particles. Concentrations from samples with glitter reached up to 14,474 μg/l Al ions; samples without glitter contained on average 1260 μg/l. A small proportion of the Al ions may originate from the alloys of the wires.

**Conclusions:**

It should be investigated whether the aluminium concentration can lead to health risks for humans. In view of the findings, orthodontists should not offer appliances containing glitter in order to minimize aluminium uptake with saliva. It needs to be clarified whether the conditions found in the oral cavity lead to the same results as under the abovementioned conditions. Legislation should be developed to limit the release of aluminium from orthodontic products.

## Einführung

In der modernen Kieferorthopädie werden zur Behandlung von Zahnfehlstellungen und Kieferdeformitäten häufig herausnehmbare Apparaturen eingesetzt. Nach Abschluss der Behandlung werden herausnehmbare Apparaturen auch als Stabilisatoren eingesetzt, um das Behandlungsergebnis zu konservieren. Um die Therapietreue der Kinder zu verbessern und somit eine möglichst lange tägliche Tragedauer der Apparatur zu gewährleisten, werden Glitzerpartikel, die zu ca. 80 Gewichts-% aus Aluminium (Al) bestehen, in verschiedenfarbige Kunststoffe einpolymerisiert. Eine tägliche Tragedauer von bis zu 16 h während einer Behandlungsperiode von 2 oder 3 Jahren wird empfohlen, um das Therapieziel zu erreichen. Ziel dieser Studie war es Aluminiumionen nachzuweisen, die bei täglicher Anwendung aus dem Kunststoff der Apparatur in den Patientenspeichel diffundieren.

Aus heutiger Sicht der Wissenschaft wird als Hauptaufnahmequelle von Aluminium die tägliche Nahrung angesehen, aber auch Kochutensilien wie Pfannen, Campinggeschirr und Konserven tragen zur täglichen Aufnahmemenge bei [1, 2]. Im weitaus geringeren Maß beeinflusst das Aluminiumvorkommen aus der Umwelt (Luft, Metallindustrie, Grundwasser) die Aufnahmemenge, die beim mitteleuropäischen Erwachsenen zwischen 1,6 mg/d und 13 mg/d und bei Kindern im Alter von 4 bis 18 Jahren bei 1,7 mg pro kg Körpergewicht (KG) und Woche liegt. Kosmetische Produkte wie aluminiumhaltige Antitranspirante, die vormals im Verruf standen, Brustkrebs zu verursachen, wurden vom Bundesinstitut für Risikobewertung (BfR) im Jahr 2020 jedoch als unkritisch und nicht kanzerogen eingestuft [3]. Auch pharmazeutische Produkte, wie Impfstoffe, enthalten mehrheitlich aluminiumhaltige Adjuvanzien, die zur Stimulation der Immunreaktion dienen [4].

Die allgemeine Studienlage zur neurotoxischen Wirkung lässt am Mausmodell erkennen, dass die Gehirnentwicklung von Jungtieren gemindert wird, nicht belegt sind jedoch Fälle von neurodegenerativen Erkrankungen beim Menschen, wie z. B. der Alzheimerkrankheit durch Aluminium. Lediglich bei Dialysepatienten kann es durch die Verwendung von aluminiumhaltiger Dialyseflüssigkeit zur sogenannten Dialyseenzephalopathie kommen [5]. In-vitro-Studien konnten nur bei sehr hohen Konzentrationen von Aluminium einen genotoxischen Effekt zeigen [1].

Obwohl bereits gezeigt wurde, dass festsitzende kieferorthopädische Apparaturen nur geringfügig zur täglichen Exposition von Aluminium beitragen können [6] und die Bioverfügbarkeit von aufgenommenen Ionen bei ca. 0,1 % liegt (Anteil der unverändert im systemischen Kreislauf zur Verfügung stehenden Ionen; [1]), sollte der Einfluss einer denkbaren Migration von Aluminiumionen in die Mundschleimhaut auch bei herausnehmbaren Apparaturen kritisch berücksichtigt werden, da es sich um eine vermeidbare und unnütze Belastung handelt.

## Material und Methoden

**Figure 1:**
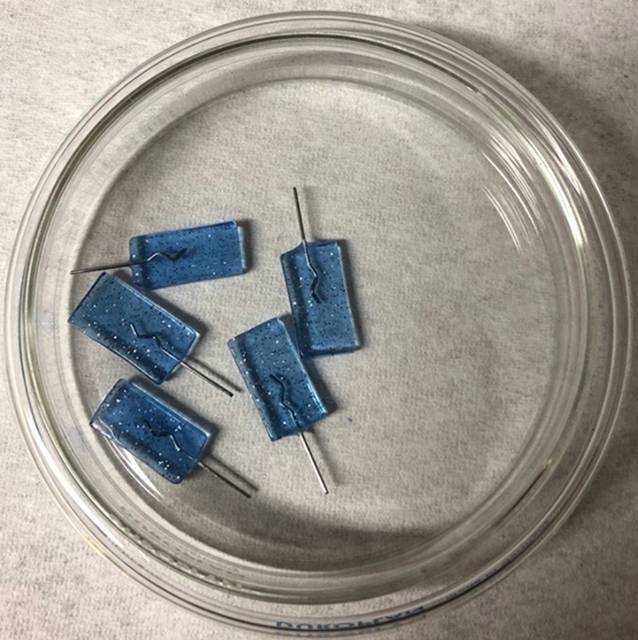

Zur Herstellung der Prüfkörper (Abb. 1) wurden 3 verschiedene Stahllegierungen, wie sie für den alltäglichen kieferorthopädischen Behandlungsablauf verwendet werden, ausgewählt: 1) Kobalt-Chrom-Nickel (Elgiloy®, Rocky Mountain Orthodontics®, Denver, USA; Remaloy®, Dentaurum, Ispringen, Deutschland), 2) Manganstahl (Noninium®, Dentaurum, Ispringen, Deutschland; Menzanium®, Scheu Dental, Iserlohn, Deutschland) und 3) Chrom-Nickel-Molybdän (Stainless Steel Wire, American Orthodontics, Sheboygan, USA; Remanium®, Dentaurum, Ispringen, Deutschland). Jede Legierung wurde durch jeweils 2 Drähte von 2 verschiedenen Herstellern repräsentiert (Tab. 1).

**Table 1:** 

Stahllegierung	Produktname	Hersteller	Zusammensetzung in %									
			C	Si	Mn	Cr	Mo	Ni	P	S	Fe	Co
Kobalt-Chrom-Nickel	Elgiloy®	Rocky Mountain Orthodontics® (Denver, USA)	0,15	–	1,5–2,5	19–21	7	14–16	–	–	Rest	39–41
	Remaloy®	Dentaurum (Ispringen, Deutschland)	0,03	< 0,5	< 0,1	18–22	3–5	19–23	–	< 0,1	4–6	Rest
Chrom-Nickel-Molybdän	Remanium®	Dentaurum (Ispringen, Deutschland)	0,05–0,15	< 2	< 2	16–19	< 0,8	6,0–9,5	< 1	< 1	Rest	–
	Stainless Steel Wire	American Orthodontics (Sheboygan, USA)	0,0–1,2	0–2	0–2	11,5–20	0,0–6,5	0–15	–	–	Rest	–
Manganstahl	Noninium®	Dentaurum (Ispringen, Deutschland)	< 0,1	< 1	16–20	16–20	1,8–2,5	< 0,2	–	0,05	Rest	–
	Menzanium®	Scheu Dental (Iserlohn, Deutschland)	0,1	1	16–20	16–20	1,6–2,5	0,2	0,05	0,05	Rest	–

*C* Kohlenstoff, *Cr* Chrom, *Co* Kobalt, *Fe* Eisen, *Mn* Mangan, *Mo* Molybdän, *Ni* Nickel, *P* Phosphor, *S* Schwefel, *Si* Silizium

5 Stücke jedes Drahttyps wurden nach den Anweisungen des Herstellers in einen kieferorthopädischen Kunststoff eingebettet. Dieser wurde zuvor teilweise eingefärbt oder mit Glitzer versehen, sodass 4 verschiedene Varianten vorlagen: transparent, transparent/Glitzer, blau und blau/Glitzer (Orthocryl® und Orthocryl ®Disco-Glimmer, Dentaurum, Ispringen, Deutschland). Es wurden rechteckige Silikonschablonen mit der Größe von 10 × 2,75 × 20 mm verwendet, um reproduzierbare Abmessungen für alle Prüfkörper zu erreichen. Jeder Prüfkörper hatte eine Gesamtfläche von 5,65 cm^2^.

Das Korrosionsmedium wurde in Anlehnung an die DIN EN ISO 10271 [7] für metallische Werkstoffe unter Verwendung von Milchsäure (PanReac, AppliChem, Darmstadt, Deutschland), Natriumchlorid (Emsure®, Merck, Darmstadt, Deutschland) und entionisiertem Wasser (Aqua B. Braun, Ecotainer®, Melsungen, Deutschland) hergestellt. Eine Gruppe von 5 Prüfkörpern wurde in eine Petrischale (Duroplan®, Schott, Mainz, Deutschland) gegeben, dann mit 50 ml des Korrosionsmediums bedeckt, bevor sie in einer Klimakammer (VEM 03/400, Heraeus Vötsch, Hanau, Deutschland) bei einer Temperatur von 37 °C für 7 Tage gelagert wurde. Parafilm® (Bemis Company, Neenah, USA) wurde verwendet, um die Petrischalen abzudecken und die Verdunstung während der Testzeit zu verhindern. Als Referenzlösung wurden 50 ml des Korrosionsmediums ohne Prüfkörper in eine Petrischale gegeben und in der Klimaprüfkammer gelagert. Nach 7 Tagen wurden 2 Proben von je 20 ml aus jeder Petrischale in Schnappdeckelgläser (Carl Roth®, Karlsruhe, Deutschland) pipettiert und mit einem Kunststoffdeckel abgedeckt. Die Menge der Aluminiumionen in den Referenzlösungen wurde mit der Menge der Aluminiumionen verglichen, die in den verschiedenen Korrosionsmedien gefunden wurden, die die Proben mit und ohne Glitzerpartikel enthielten. Darüber hinaus verglichen wir die Menge an Aluminiumionen in den Lösungen der Prüfkörper, die Glitzerpartikel enthielten, mit denen, die keine Glitzerpartikel enthielten.

Alle Proben wurden mittels Massenspektrometrie mit induktiv gekoppeltem Plasma (ICP-MS 7700 Serie, Agilent Technologies, Santa Clara, USA) auf Aluminium und andere Metallionen analysiert.

## Ergebnisse

Der durchgeführte Kolmogorow-Smirnow-Test ergab, dass für die Al-Konzentrationen in den verschiedenen Testlösungen keine Gauß’sche Normalverteilung vorliegt. Daher wurde anschließend ein Kruskal-Wallis-Test zur statistischen Analyse als nichtparametrischer Test angewandt. Ein α = 0,05 wurde als Hinweis für statistische Signifikanz angesehen.

**Figure 2:**
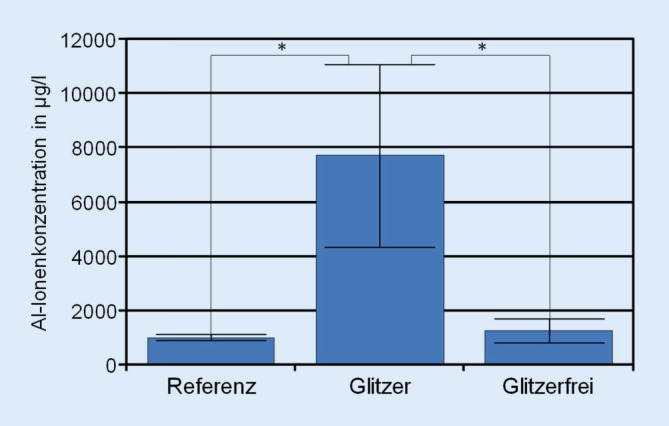

Die Konzentration von Aluminiumionen im Korrosionsmedium von Prüfkörpern mit Glitzerpartikeln war signifikant höher als die der Referenzproben (Abb. 2). Im Mittel erreichten sie Konzentrationen um 7722 μg/l. Im Vergleich dazu wiesen die Referenzlösungen im Mittel Konzentrationen um 983 μg/l auf. Eine zweite Referenzlösungsmessreihe mit einer neuen Charge demineralisierten Wassers ergab Al-Referenzwerte von 4,8 µg/l. Die Lösungen, die Prüfkörper ohne Glitzerpartikel enthielten, zeigten im Durchschnitt etwas erhöhte Konzentrationen um 1260 μg/l. Somit zeigten glitzerhaltige Prüfkörper signifikant höhere Aluminiumkonzentrationen als glitzerfreie Prüfkörper und Referenzlösungen. Die glitzerfreien Prüfkörper lagen lediglich geringfügig über den gemessenen Werten der Referenzlösungen.

Sämtliche Messwerte der Einzelproben sind in Tab. 2 zusammengefasst.

**Table 2:** 

Drahthersteller, Produktname	Kunststoffmerkmale	Konzentration Al-Ionen 1. Probe (μg/l)	Konzentration Al-Ionen 2. Probe (μg/l)
Dentaurum, Remanium®	Farblos	2381	2444
	Farblos Glitzer	5953	5991
	Blau	1119	1105
	Blau Glitzer	4121	4104
American Orthodontics, Stainless Steel Wire	Farblos	1573	1660
	Farblos Glitzer	5100	5800
	Blau	1649	1655
	Blau Glitzer	11.612	11.604
Dentaurum, Noninium®	Farblos	1128	1107
	Farblos Glitzer	10.061	9625
	Blau	935	927
	Blau Glitzer	4022	4084
Scheu, Menzanium®	Farblos	1046	1073
	Farblos Glitzer	5584	5673
	Blau	933	996
	Blau Glitzer	11.947	11.785
Rocky Mountain Orthodontics®, Elgiloy®	Farblos	1204	1279
	Farblos Glitzer	8870	8883
	Blau	1005	994
	Blau Glitzer	4070	3981
Dentaurum, Remaloy®	Farblos	1043	1006
	Farblos Glitzer	7404	7442
	Blau	994	994
	Blau Glitzer	14.474	13.112
Referenzen	Ohne Prüfkörper 1.1	1056	1027
	Ohne Prüfkörper 1.2	1030	881
	Ohne Prüfkörper 2.1	980	944
	Ohne Prüfkörper 2.2	884	1063
	Ohne Prüfkörper – neue Charge demineralisiertes Wasser	4,8	–

## Diskussion

Die Ergebnisse zeigen, dass die hohe Konzentration von Aluminiumionen hauptsächlich durch die Glitzerpartikel in den jeweiligen Prüfkörpern verursacht wird. Ein geringer Anteil der festgestellten Aluminiumkonzentration könnte auf einen Al-Gehalt in den Legierungen der Drähte zurückzuführen sein, obwohl Aluminium nicht als Legierungsbestandteil erwähnt wird. Wie Tab. 2 zeigt, ist der Einfluss der verschiedenen Drahtmaterialien auf die Aluminiumkonzentration jedoch gering und zeigt auch eine geringe Varianz, was die Zusammenfassung zu einem gemeinsamen Mittelwert rechtfertigt. Die Aluminiumkonzentration um 983 μg/l in der Referenzgruppe kann durch vorübergehende Spuren von Aluminium aus dem demineralisierten Wasser, das die Laborspülmaschine versorgt, verursacht worden sein. Dieses Wasser wurde vor Beginn des Versuchs zur Reinigung der Petrischalen verwendet.

Um nachzuweisen, dass Aluminium definitiv aus den Glitzerpartikeln freigesetzt wird und nicht aus möglicherweise kontaminiertem demineralisierten Wasser aus der Spülmaschine stammt, wurde eine zweite Testreihe mit nur einer Gruppe von 5 glitzerhaltigen Prüfkörpern durchgeführt. Eine Kontamination der verwendeten Chemikalien und einer neuen Charge des benutzten demineralisierten Wassers wurde durch eine vorherige ICP-MS-Analyse ausgeschlossen. In diesem zweiten Referenzlösungstest resultierten eine vernachlässigbar geringe Aluminiumkonzentration von 4,8 μg/l und damit sehr geringe Al-Blindwerte. Die Aluminiumkonzentration in den Lösungen der Glitzerprüfkörper lag jedoch wiederum bei etwa 8800 μg/l. Damit konnte erneut bestätigt werden, dass die hohe Aluminiumkonzentration aus den glitzerhaltigen Prüfkörpern herrührt.

Die hohe Varianz der Aluminiumkonzentration in den glitzerhaltigen Proben von 3981 μg/l bis zu 14.474 μg/l lässt sich mit der Menge und der Position der Glitzerpartikel im Kunststoff der Prüfkörper erklären. Die Autoren vermuten, dass Proben mit vielen Glitzerpartikeln nahe der Oberfläche der Kunststoffprüfkörper mehr Ionen in das Korrosionsmedium abgeben. Obwohl die Glitzerpartikel vor der Herstellung der Prüfkörper gut mit dem Monomerpulver vermischt wurden, kann zudem nicht garantiert werden, dass alle Prüfkörper die gleiche Menge an Aluminium enthalten. Insbesondere die Tatsache, dass Zahntechniker Glitzer und Pulver in unterschiedlichen Mischungsverhältnissen mischen, kann zu noch höheren Konzentrationen von Aluminium in der Mundhöhle führen. Zukünftige Versuche sollen den individuellen Einfluss des Drahtes, des pH-Wertes und der Inhomogenität der Glitzerpartikel untersuchen.

Im Hinblick auf die individuelle Größe des Kiefers eines Patienten kann davon ausgegangen werden, dass größere Apparaturen, die dementsprechend mehr Kunststoff inkl. Glitzer enthalten, noch mehr Aluminium an den Speichel abgeben. Die durchschnittliche Oberfläche der bimaxillären kieferorthopädischen Apparatur eines Teenagers kann in Abhängigkeit von der individuellen Größe und Form des Kiefers auf etwa 48 cm^2^ geschätzt werden. Bezüglich der durchschnittlichen Aluminiumfreisetzung glitzerhaltiger Apparaturen in den von uns durchgeführten Testreihen (7722 μg/l) können wir als wöchentliche zusätzliche Aluminiumbelastung durch eine Plattenapparatur eine Menge von 437 μg berechnen. Diese Menge ergibt sich durch Skalierung der Oberfläche unserer 5 Prüfkörper je Petrischale (insgesamt 28,25 cm^2^) auf die Größe einer typischen Plattenapparatur und die Umrechnung der Aluminiumkonzentration in das Korrosionsvolumen von 50 ml auf die insgesamt abgegebene Aluminiummasse.

Höchstwahrscheinlich erhöht der niedrige pH-Wert der Testlösung von 2,3, der durch die DIN EN ISO 10271 für statische Immersionstests gefordert wird [7], die Menge der in die Lösung diffundierenden Aluminiumionen im Vergleich zum durchschnittlichen pH-Wert von 6,5 des menschlichen Speichels. Dieser unterliegt allerdings nach der Aufnahme von sauren Getränken (z. B. Limonaden, Säften) oder süßen Speisen einem pH-Abfall bis das im Speichel enthaltene Puffersystem zur Neutralisierung führt. Vorliegende Studien konnten bereits die Migration von Aluminium und anderen Ionen in umliegende Mukosazellen darlegen [8–11]. In Tierversuchen zeigten epitheliale Zellen des Verdauungstraktes von Mäusen nach erhöhter oraler Aufnahme von Aluminium histologisch klare Zeichen von Entzündungsreaktionen und erhöhter interzellulärer Permeabilität [10]. Zukünftige Untersuchungen sollen zeigen, ob die Aluminiumionen kontinuierlich auch über einen Zeitraum von 7 Tagen hinaus abgegeben werden oder ob es zu einer Stagnation der Abgabe kommt. Wir möchten betonen, dass es mehrere andere Hersteller gibt, die ähnliche Produkte mit den gleichen Inhaltsstoffen unter Bezugnahme auf das Sicherheitsdatenblatt anbieten. Es ist mehr als wahrscheinlich, dass diese Produkte zu ähnlichen Ergebnissen führen.

## Schlussfolgerung

In dieser Studie wurde die Konzentration von Al-Ionen, die aus Prüfkörpern mit Aluminiumglitzerpartikeln über einen Zeitraum von 7 Tagen in das Korrosionsmedium freigesetzt wurden, im Vergleich zu Prüfköpern, die keine Aluminiumglitzerpartikel enthielten, untersucht und verglichen. Es wurde eine signifikant höhere Konzentration von Al-Ionen von bis zu 14.474 μg/l in der Lösung von Prüfkörpern mit Glitzerpartikeln gefunden. Diese hohe Aluminiumkonzentration scheint durch die Glitzerpartikel verursacht zu werden, die aus Aluminium (80 Gew.-%) und Epoxid (20 Gew.-%) bestehen. Es ist zu diskutieren, ob eine junge und sensitive Bevölkerungsgruppe mit glitzerhaltigen herausnehmbaren kieferorthopädischen Apparaturen versorgt und so eine zusätzliche Al-Belastung der jungen Patienten verursacht werden sollte. Auch wenn sich die aufgenommene Menge Aluminium unterhalb des von der JECFA (Joint FAO/WHO Expert Committee On Food Additives) im Jahr 2012 empfohlenen PTWI-Werts (Provisional Tolerable Weekly Intake) von 2 mg pro kg Körpergewicht und Woche und auch unterhalb des TWI-Werts der EFSA (European Food Safety Authority) aus 2008 von 1 mg pro kg Körpergewicht und Woche bewegt, herrscht im oralen Milieu eine erhöhte Konzentration in direkter Umgebung zur Mundschleimhaut, die kritisch zu bewerten ist. Diese spezifische Exposition sollte zusätzlich zur täglichen Aufnahme von Aluminium über die gesamte Behandlungsdauer überprüft werden.

Die Autoren sind aus folgenden Gründen der Meinung, dass gerade bei empfindlichen Bevölkerungsgruppen wie Kindern und Jugendlichen zusätzliche Belastungen mit Aluminium vermieden werden sollten:

1. Es handelt sich um vermeidbare und „unnütze“ Belastungen, da das Aluminium nur zu Zwecken der Therapietreue oder aus kosmetischen Gründen in den Kunststoff eingebracht wird. Hier könnte eine Diskussion um technologische Verbesserung angestoßen werden, wie man z. B. über ein anderes Herstellungsverfahren die Glitzerpartikel im Innern der Kunststoffkörper positionieren könnte und dadurch keine nennenswerte Migration stattfinden würde.
2. Es handelt sich nicht „nur“ um eine orale Aufnahme, welche man mit Hintergrundbelastungen vergleichen müsste, sondern um langfristige Belastungen über teils mehrere Jahre, wobei vor allem empfindliche Gewebe (wie die Mundschleimhaut) exponiert sind.
3. Eine bereits vorhandene Hintergrundbelastung kann nicht zwingend das Argument gegen Belastungsminimierungen sein.

## References

[1] Aguilar F, Autrup H, Barlow S (2008). Scientific opinion of the panel on food additives, flavourings, processing aids and food contact, materials on a request from European Commission on safety of aluminium from dietary intake. EFSA J.

[2] Weidenhamer JD, Fitzpatrick MP, Biro AM (2017). Metal exposures from aluminum cookware: an unrecognized public health risk in developing countries. Sci Total Environ.

[3] Bundesinstitut für Risikobewertung (BfR) (2020). Neue Studien zu aluminiumhaltigen Antitranspirantien: Gesundheitliche Beeinträchtigungen durch Aluminium-Aufnahme über die Haut sind unwahrscheinlich.

[4] Wang ZB, Xu J (2020). Better adjuvants for better vaccines: progress in adjuvant delivery systems, modifications, and adjuvant-antigen codelivery. Vaccines.

[5] Tietz T, Lenzner A, Kolbaum AE (2019). Aggregated aluminium exposure: risk assessment for the general population. Arch Toxicol.

[6] Olszewska A, Hańć A, Barałkiewicz D, Rzymski P (2019). The contribution of orthodontic braces to aluminum exposure in humans: an experimental in vitro study. Environ Sci Pollut Res.

[7] EN ISO 10271 (2020). Proposal for corrosion evaluation of orthodontic brackets and wires per EN ISO 10271 dentistry—corrosion test methods for metallic materials.

[8] Sajnóg A, Hanć A, Koczorowski R, Barałkiewicz D (2017). New procedure of quantitative mapping of Ti and Al released from dental implant and Mg, Ca, Fe, Zn, Cu, Mn as physiological elements in oral mucosa by LA-ICP-MS. Talanta.

[9] Loyola-Rodríguez JP, Lastra-Corso I, García-Cortés JO (2020). In vitro determination of genotoxicity induced by brackets alloys in cultures of human gingival fibroblasts. J Toxicol.

[10] Jeong CH, Kwon HC, Kim DH (2020). Effects of aluminum on the integrity of the intestinal epithelium: an in vitro and in vivo study. Environ Health Perspect.

[11] Martín-Caméan A, Jos A, Puerto M (2015). In vivo determination of aluminum, cobalt, chromium, copper, nickel, titanium and vanadium in oral mucosa cells from orthodontic patients with mini-implants by Inductively coupled plasma-mass spectrometry (ICP-MS). J Trace Elem Med Biol.

